# Challenges and Enhancing Strategies of Equine Semen Preservation: Nutritional and Genetic Perspectives

**DOI:** 10.3390/vetsci12090807

**Published:** 2025-08-25

**Authors:** Abd Ullah, Wenting Chen, Limeng Shi, Menghan Wang, Mingyang Geng, Jincheng Na, Muhammad Faheem Akhtar, Muhammad Zahoor Khan, Changfa Wang

**Affiliations:** 1 College of Agriculture and Biology, Liaocheng University, Liaocheng 252000, Chinazahoorkhan@lcu.edu.cn (M.Z.K.); 2 Yili Kazak Autonomous Prefecture Livestock General Station, Xinjiang Autonomous Region, Yili 835000, China

**Keywords:** equine semen, preservation challenges, enhancing strategies, nutritional supplements, potential genes

## Abstract

Equine semen preservation is fundamental to modern equine reproduction, supporting breeding programs, genetic conservation, and industry sustainability. However, significant challenges persist, including temperature sensitivity, oxidative stress, bacterial contamination, individual variability, and lack of standardized preservation protocols. These factors contribute to reduced sperm viability and fertility following cryopreservation. This review examines critical obstacles in equine semen preservation, focusing on cryopreservation sensitivity, molecular damage mechanisms, economic constraints, and seasonal quality variations. We analyze the molecular and structural alterations (e.g., oxidative stress, membrane damage, and DNA fragmentation) and their impact on cryopreservation success. The review evaluates evidence-based enhancement strategies, including nutritional supplementation and genetic approaches, for improving semen quality. Nutritional interventions that utilize antioxidants, polyunsaturated fatty acids (PUFAs), and nutraceuticals have demonstrated promising results in enhancing sperm motility, preserving membrane integrity, and improving overall semen quality. Additionally, we discuss key candidate genes associated with equine semen-quality traits, including sperm motility, viability, and cryotolerance. The integration of nutritional supplementation and genetic selection strategies presents viable pathways for optimizing equine semen preservation techniques. These combined approaches offer potential solutions for overcoming current limitations, ultimately supporting sustainable breeding programs and advancing genetic conservation efforts in the equine industry.

## Simple Summary

This comprehensive review examines the complex challenges facing equine semen preservation, with a particular emphasis on how stallion sperm is uniquely vulnerable to damage during cryopreservation compared to other livestock species. The study identifies major obstacles, including high individual variability between stallions and bacterial contamination, as well as the molecular and structural damage that occurs when stallion sperm undergoes freezing, including oxidative stress, membrane instability, and DNA fragmentation, which compromises reproductive success. The authors evaluate two primary improvement strategies: targeted nutritional supplementation with antioxidants, vitamins, and omega-3 fatty acids that enhance sperm membrane integrity and motility parameters. Additionally, the research explores genetic approaches by identifying specific candidate genes like *NME8*, *OR2AP1*, and *CRISP3* that are associated with improved cryotolerance and sperm-quality traits. The integration of these nutritional, genetic methodologies and advances in extender formulations offers promising pathways to overcome current limitations and optimize artificial insemination programs in the equine industry.

## 1. Introduction

Equines represent a critical component of global biodiversity and agricultural systems, providing multifaceted contributions, including livestock, agricultural resources, sports animals, landscape management, and leisure companions [1]. The preservation of equine populations has emerged as a significant concern at the nexus of conservation imperatives and expanding commercial demands [2,3,4]. Enhancing reproductive performance in equines requires a multifaceted approach that combines traditional breeding practices with modern reproductive technologies to improve fertility rates, foal survival, and genetic management for sustainable population maintenance [5,6,7]. Growing needs drive studies enhancing equine reproduction for sustainable populations.

Central to these optimization efforts is a comprehensive understanding of the genetic determinants underlying economically significant traits, with particular emphasis on reproductive parameters. Semen, a complex biological fluid comprising spermatozoa suspended in glandular secretions, constitutes the fundamental male reproductive contribution essential for fertilization [8]. The quality and viability of semen directly impact reproductive efficiency across equine species [9]. Consequently, equine semen holds profound biological, genetic, and economic significance across diverse domains, from selective breeding programs to fundamental scientific research [10,11]. Thus, optimizing semen quality and preservation is key to improving stallion fertility and advancing equine breeding.

The systematic evaluation of semen quality represents a crucial component in assessing a stallion’s reproductive potential and diagnosing instances of subfertility or infertility [12]. Such evaluations provide critical insights into breeding capabilities and facilitate the identification of reproductive abnormalities [13]. The cryopreservation of stallion semen offers substantial advantages, including reduced disease transmission risk, elimination of geographical constraints, and indefinite preservation of valuable genetic material [14]. Therefore, advancing research methodologies and refining techniques for semen evaluation and preservation are imperative for maximizing genetic potential and reproductive success in stallions, ultimately supporting the sustainable improvement of equine populations.

However, maintaining optimal semen quality presents significant challenges due to the inherent sensitivity of equine sperm to handling procedures, temperature fluctuations, and preservation protocols [15,16]. These preservation challenges primarily derive from the distinctive physiological characteristics of equine spermatozoa. Factors such as oxidative stress [17], temperature sensitivity [18], and the complex structural organization of sperm membranes significantly contribute to diminished motility, viability, and fertility during storage [19]. Furthermore, cryopreservation processes involving freezing and subsequent thawing exacerbate cellular damage through ice crystal formation and osmotic stress, potentially compromising functional integrity [20].

Contemporary collection methodologies predominantly utilize specialized artificial vagina systems, including the Colorado and Missouri models [21,22]. While lubricants facilitate this collection process, their potentially deleterious effects on semen quality necessitate careful consideration [23]. Following collection, comprehensive quality assessment encompasses parameters, including volume, concentration, motility, and morphological normality. Supplementary analytical procedures—such as acrosomal and membrane integrity evaluation, hypo-osmotic swelling tests (HOST), mitochondrial activity assessment, and thermal resistance analysis—provide a detailed characterization of semen quality [24].

Current preservation methodologies include refrigerated storage at 5 °C (maintaining viability for up to 72 h) and cryopreservation, each presenting distinct challenges. The efficacy of refrigerated storage can be compromised by factors, including seminal plasma concentration, metabolic waste accumulation, and membrane integrity deterioration [25,26]. Cryopreservation protocols typically incorporate protective agents, such as centrifuged egg yolk (CEY) and N, N-dimethylformamide, with subsequent controlled cooling and storage in liquid nitrogen. Despite these established protocols, preservation efficacy remains influenced by cooling rates, cryoprotectant concentrations, and handling techniques, potentially resulting in diminished post-thaw quality and fertility [27,28].

While traditional preservation methods have demonstrated considerable utility, persistent challenges, including individual variation, osmotic stress, and suboptimal cryoprotectant formulations, highlight the need for continued refinement. Molecular investigations exploring key mutations and genomic regions influencing semen-quality traits represent a promising frontier in addressing these limitations [11]. This review comprehensively examines the principal challenges associated with equine semen preservation and critically evaluates contemporary strategies designed to enhance semen quality and reproductive outcomes.

## 2. Methodology for Literature Search

This review systematically explores the key challenges in equine semen preservation and critically assesses modern strategies aimed at improving semen quality and reproductive efficiency. To ensure a comprehensive and systematic evaluation of current knowledge, a structured literature search was conducted across four major databases: PubMed, Web of Science, Scopus, and Google Scholar. The search targeted peer-reviewed articles published between 2005 and 2025. The relevant literature was identified using a combination of Boolean operators and specific keywords, including equine semen, semen preservation, stallion fertility, nutritional supplementation, oxidative stress, sperm cryopreservation, and genetic markers. Reference lists of key articles were also manually screened to identify additional relevant publications.

**Figure 1:**
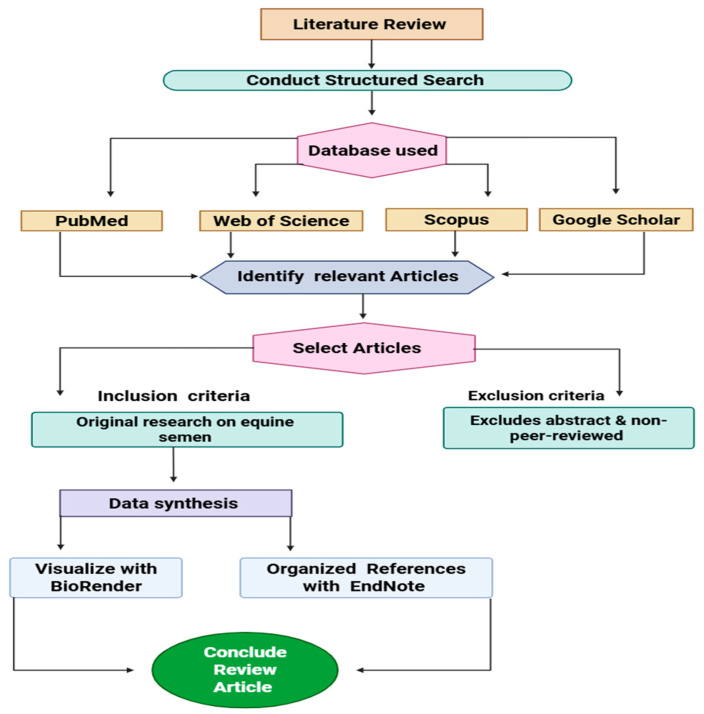
A flowchart of the literature search and articles selection.

Articles were selected based on predefined inclusion and exclusion criteria to ensure scientific rigor. Inclusion criteria encompassed original research articles focused on equine semen quality, fertility, preservation techniques, and enhancement strategies through nutritional or genetic interventions. A total of 350 records were initially retrieved, but after the screening, only 200 were cited, and only studies published in English were considered to ensure clarity and consistency in interpretation. Furthermore, exclusion criteria involved conference abstracts, book chapters, unpublished data, and non-indexed or non-peer-reviewed sources. This approach ensured the reliability and reproducibility of the review while maintaining relevance to the scope of equine reproductive science. Data synthesis included a visual representation of key concepts using BioRender for professional scientific illustrations. References were organized and formatted using EndNote to ensure accuracy and consistency. For ease of reference, the literature search methodology and article inclusion and exclusion criteria for the current review are provided in Figure 1.

## 3. Challenges in Equine Semen Preservation

### 3.1. Equine Sperm Variability

**Figure 2:**
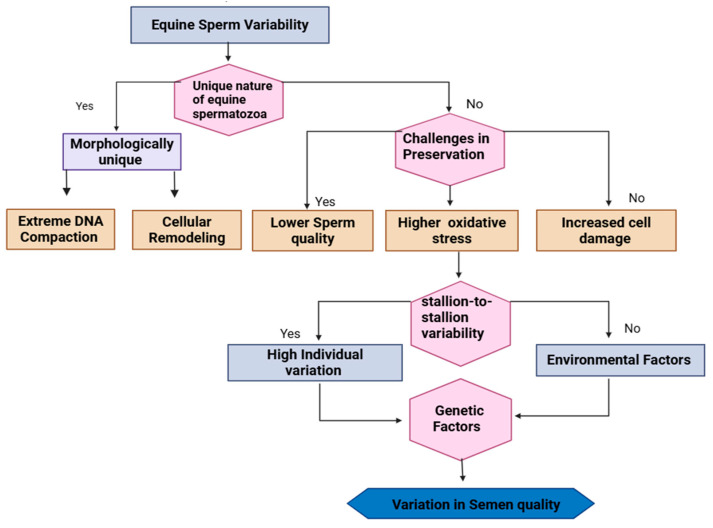
The species-specific factors contributing to equine semen preservation complexity. The flowchart shows the unique characteristics of equine reproduction that complicate semen preservation. The schematic representation shows that sperm variability and morphological uniqueness (extreme DNA compaction and cellular remodeling) and preservation-specific challenges (oxidative susceptibility and cellular damage) affect semen quality. Individual stallion variation and environmental factors are shown as additional variables contributing to the high degree of variability in the preservation of semen quality.

There are several challenges to equine semen preservation due to the unique nature of equine semen [19]. Equine spermatozoa are morphologically unique when compared to other mammalian cells, having undergone extreme DNA compaction and cellular remodeling, resulting in the absence of the majority of the cytoplasm and organelles that are present in somatic cells [29]. However, stallion sperm preservation still poses challenges compared with other species, with overall lower sperm quality, higher rates of oxidative stress, and generally increased cell damage [30]. One of the challenges for those attempting to cryopreserve stallion spermatozoa is dealing with the stallion-to-stallion variability in the cryosurvival of their semen [31]. Stallions have a high individual variation that depends on both environmental and genetic factors [31]. There is a wide variation in semen quality between individuals, which may reflect variations in the antioxidant properties of their seminal plasma [32]. This variability in equine semen, influenced by both genetic and environmental factors and closely associated with differences in seminal plasma antioxidant properties, is depicted in the schematic representation in Figure 2.

### 3.2. Biological Challenges

**Figure 3:**
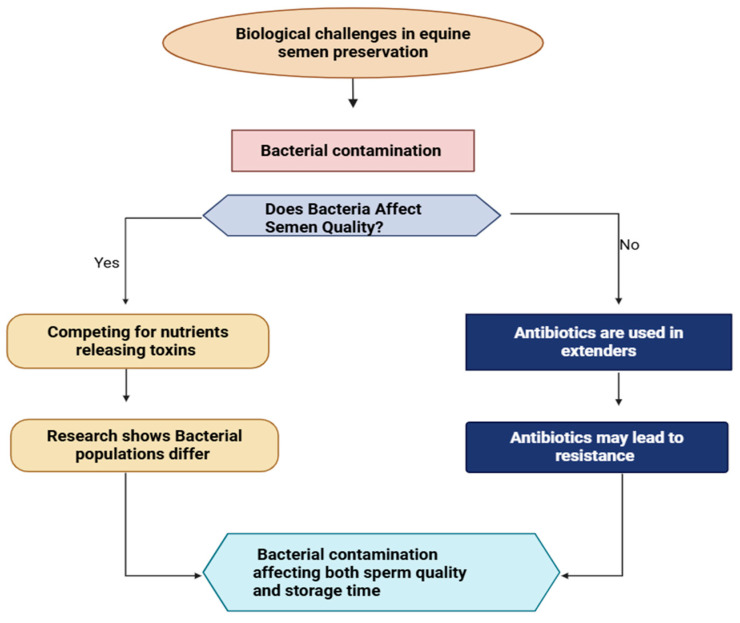
Bacterial contamination management in equine semen processing. The flowchart illustrates the dual challenge of bacterial contamination in equine semen preservation. The flowchart presents two scenarios: when bacteria affect semen quality (leading to nutrient competition and toxin production) and when antibiotics are used prophylactically. Both pathways ultimately impact preservation success, highlighting the complex balance between controlling contamination and avoiding antibiotic resistance while maintaining sperm viability during storage.

Bacterial contamination is a major obstacle in equine semen preservation, compromising sperm quality and storage viability (Figure 3). Bacteria colonizing the male reproductive tract can contaminate semen during collection, competing for nutrients, producing harmful metabolic byproducts, and releasing toxins that damage sperm [33]. Although antibiotics are commonly used in semen extenders to suppress bacterial growth, they often fail to eliminate contamination and may contribute to antimicrobial resistance [33,34]. Studies reveal significant variability in bacterial populations between stallions and even among ejaculates from the same breeding facility, emphasizing the complex microbial dynamics in semen [34]. Bacterial nutrient competition, toxin release, and their final assault on sperm vitality are visualized in the flowchart in Figure 3.

Beyond impairing sperm integrity, bacterial contamination can reduce fertility in inseminated mares and diminish semen longevity during storage [35]. Research across multiple countries, including Spain [36], Italy [37], Germany [38], Portugal [39], and Austria [40], has documented the prevalence of bacterial contamination in stallion semen, highlighting its global significance. These findings underscore the need for improved antimicrobial strategies to enhance semen preservation while mitigating resistance risks.

### 3.3. Equine Semen Sensitivity to Cryopreservation

Cryopreservation poses significant challenges for stallion semen due to its high sensitivity to temperature fluctuations and osmotic stress. Unlike cattle, where frozen semen is widely used in artificial insemination, equine breeding relies more on cooled semen due to poor post-thaw sperm quality and fertility rates [19]. Stallion sperm are highly susceptible to cold shock when cooled rapidly (at a rate exceeding 0.3 °C/min), leading to membrane damage and reduced viability [41,42]. Additionally, cryopreservation also disrupts osmotic balance, making equine sperm more vulnerable than those of other species, particularly during cryoprotectant addition and removal [43].

Cryopreservation alters DNA methylation patterns and disrupts nucleoprotein structures, reducing fertilization potential [44,45]. Improper freezing rates cause ice crystal formation, mechanically damaging spermatozoa [46]. Compared to other livestock, stallions exhibit the poorest post-thaw outcomes, with only about 30% motility and viability, whereas sheep and pigs show better resilience at approximately 50% and 30%, respectively [47,48]. These limitations highlight the need for species-specific protocols to enhance post-thaw quality.

### 3.4. Oxidative Stress and Its Effect on Equine Semen Preservation Quality

**Figure 4:**
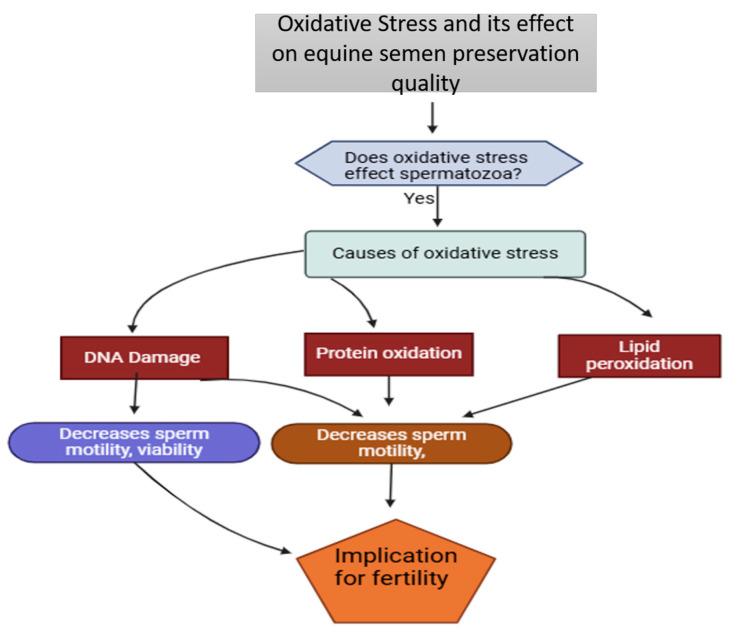
The oxidative damage pathways in equine sperm cryopreservation. The mechanistic flowchart demonstrates how oxidative stress compromises equine sperm during preservation. The diagram traces the progression from initial oxidative stress through three primary damage pathways (DNA fragmentation, protein denaturation, and membrane lipid peroxidation) to their combined effects on sperm function. Both motility and viability are shown as key parameters affected by oxidative damage, with direct implications for reproductive success.

Oxidative stress is a major contributor to sperm damage during cryopreservation [49]. Excessive reactive oxygen species (ROS) induce lipid peroxidation, protein and DNA damage, apoptosis, and mitochondrial dysfunction, ultimately reducing sperm motility, viability, and acrosomal integrity [50,51,52]. The cryopreservation process exacerbates oxidative stress by depleting natural antioxidants, such as superoxide dismutase (*SOD1*), further increasing susceptibility to oxidative damage [53,54]. Additionally, hydroxyl radicals and malondialdehyde impair mitochondrial activity and DNA integrity, leading to diminished fertility and potential developmental abnormalities in embryos derived from cryopreserved sperm [55,56]. This cumulative impact of oxidative stress on equine sperm structure and function, particularly its role in post-thaw fertility reduction, is exemplified in the mechanistic flowchart in Figure 4. The accumulation of oxidative damage in germ cells is a major concern, as it not only impairs immediate sperm function but also contributes to long-term detrimental effects on embryo development [57]. Both oxidative stress and the buildup of damaged proteins escalate during semen storage, highlighting the need for effective antioxidant strategies to mitigate these effects [57,58]. Addressing oxidative stress is crucial for enhancing sperm quality after thawing and ensuring the success of artificial reproduction techniques in equine breeding programs.

### 3.5. Limited Understanding

**Figure 5:**
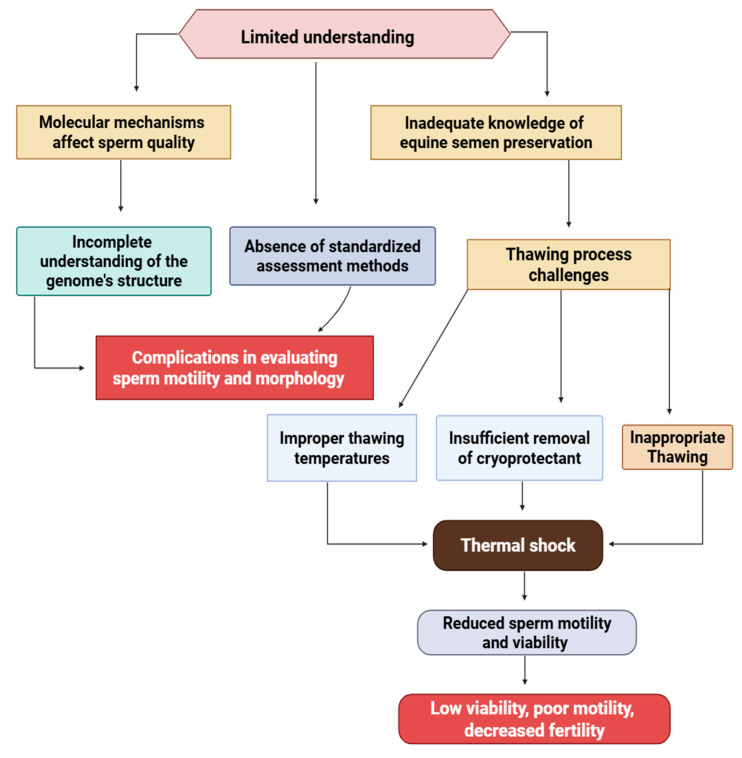
The systematic barriers to effective equine semen cryopreservation. The figure shows the cascade of challenges in equine semen preservation technology. Three primary knowledge gaps (molecular mechanisms, standardized protocols, and thawing procedures) create downstream complications that ultimately compromise preservation success. The diagram shows how insufficient understanding leads to inadequate assessment methods and improper handling techniques, resulting in reduced sperm quality and compromised fertility outcomes.

A limited understanding of the molecular mechanisms that affect sperm quality, along with the absence of standardized assessment methods, presents significant challenges for preserving equine semen. Current evaluations of stallion semen, which focus on factors such as sperm motility and morphological characteristics, are complicated by an incomplete understanding of the genome’s molecular structure and its connection to sperm development and preservation after thawing [13,59]. Moreover, the thawing process itself poses a critical challenge; improper techniques, such as incorrect thawing temperatures, insufficient removal of cryoprotectants, or inappropriate thawing rates, can result in thermal shock, cellular damage, and reduced sperm motility and viability [60,61,62]. These issues collectively lead to characteristically low post-thaw viability, impaired motility, and reduced fertility levels, as illustrated in the flowchart in Figure 5.

### 3.6. Effect of Season on Semen Quality

Seasonal variations significantly affect the quality of equine semen, posing challenges for effective preservation strategies. Semen collected during the breeding season (March to June) typically has superior motility and is more suitable for cryopreservation [63]. In contrast, samples obtained in the non-breeding season (September to December) often demonstrate reduced motility and overall quality, emphasizing the need to account for seasonal factors in preservation protocols [64].

These fluctuations are primarily driven by photoperiodic changes and the complex interplay of reproductive hormones, including melatonin, gonadotrophins, steroids, inhibin, prolactin, and endogenous opioids, which collectively regulate stallion reproductive activity [65,66]. Among the seasons, spring provides the most favorable conditions for semen preservation, yielding sperm with enhanced motility and reduced DNA damage [67]. Summer also supports effective preservation due to lower oxidative stress levels, whereas winter presents greater challenges because of increased oxidative stress [68].

Overall, the success of equine semen preservation is highly season-dependent, with spring and summer being the most optimal periods due to higher sperm concentration and improved morphometric characteristics.

### 3.7. Costs and Accessibility

Economic challenges significantly impact equine semen preservation, adding to the complexity of the process. Freezing laboratories face high costs related to producing and maintaining various freezing extenders, which are crucial for testing and optimizing semen preservation for each new stallion [35]. Additionally, the expenses associated with semen processing, like freezing and storage, are often greater than those for breeding with cooled, shipped semen. These extra costs are typically passed on to mare owners through increased or non-guaranteed breeding fees. When coupled with the more detailed and time-consuming management requirements for mares, breeding with frozen semen can become prohibitively expensive [23]. The financial strain of equine semen preservation underscores the need for cost-effective strategies; beyond these barriers, environmental conditions, osmotic stress, apoptosis, and other variables also have an impact on equine semen preservation, as detailed in Table 1.

## 4. Molecular and Structural Alterations in Equine Semen Preservation

**Figure 6:**
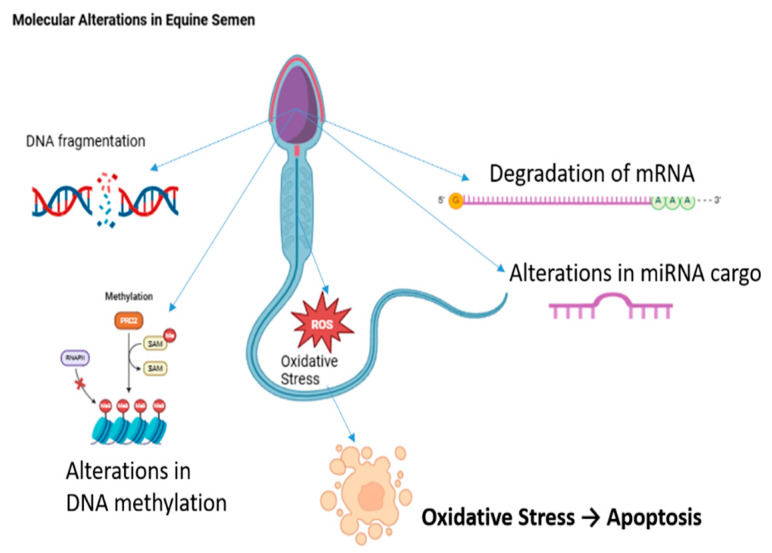
Cryopreservation induces multiple molecular disruptions that compromise sperm viability and fertilizing capacity. The figure illustrates key cryodamage mechanisms, including excessive reactive oxygen species (ROS) production leading to lipid peroxidation, protein damage, and DNA fragmentation; alterations in DNA methylation patterns; damage to sperm mRNA affecting fertilizing potential; and dysregulation of miRNA cargo critical for sperm maturation, fertilization, and early embryonic development.

Cryopreservation of equine semen is a critical process that aims to preserve sperm viability and functionality for future use. However, this process is inherently stressful for sperm cells, primarily due to the increased production of reactive oxygen species (ROS). Elevated ROS levels can create an imbalance with the sperm’s antioxidant capacity, leading to membrane lipid peroxidation (LPO), protein damage, and DNA fragmentation [77]. These oxidative stress-induced alterations not only compromise the structural integrity of sperm cells but also impair their metabolic functions. For instance, cryopreservation has been shown to alter DNA methylation patterns in equine sperm, independent of conventional semen quality parameters, such as motility and membrane integrity [45]. Additionally, cryopreservation can damage messenger RNA (mRNA) within sperm, leading to reduced fertilizing capacity in frozen-thawed semen [78]. The miRNA cargo of equine sperm, which plays essential roles in sperm maturation, fertilization, and early embryogenesis, is also affected by cryopreservation [79,80]. Changes in miRNA profiles following freezing and thawing can disrupt these critical processes, further contributing to reduced fertility outcomes [79,80]. Molecular-level disruptions, including ROS-driven DNA fragmentation, altered DNA methylation, impaired mRNA stability, and dysregulated miRNA cargo, are schematically summarized in Figure 6.

Structural alterations are another significant consequence of cryopreservation in equine sperm. The process inflicts considerable damage on spermatozoa, primarily through oxidative stress, which disrupts membrane lipids, proteins, and DNA [17,81]. Excessive ROS generation during freeze–thaw cycles triggers apoptosis via caspase activation and mitochondrial dysfunction, or in cases of severe damage, necrosis due to irreversible membrane injury [17,81]. The sperm plasma membrane’s lipid bilayer is particularly vulnerable to such damage, with lipid peroxidation leading to loss of structural integrity and increased permeability [82,83]. The high polyunsaturated fatty acid (PUFA) content in equine sperm membranes makes them particularly susceptible to oxidative damage, further compromising membrane fluidity and function [82,83].

**Figure 7:**
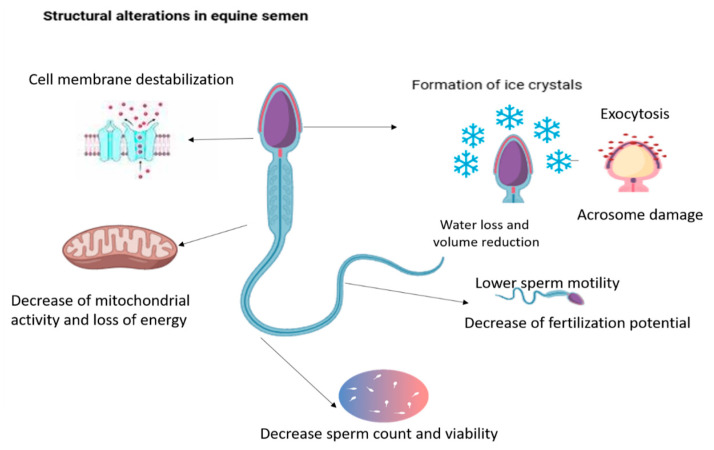
Cryopreservation induces extensive structural damage that compromises sperm integrity and motility. The figure highlights the lipid peroxidation of the plasma membrane, collapse of mitochondrial membrane potential with reduced ATP synthesis, caspase-mediated apoptosis or necrosis, disruption of flagellar and axonemal proteins, and intracellular ice crystal formation leading to acrosomal and organelle damage, collectively impairing fertilizing capacity.

Additionally, cryopreservation also induces decreased mitochondrial activity in equine spermatozoa, primarily through the cold shock-mediated reduction of mitochondrial membrane potential, which impairs ATP synthesis and compromises motility [84]. Additionally, cryopreservation reduces viable sperm concentration in equine semen due to cryodamage [85] and leads to decreased sperm motility, primarily due to structural and functional damage to the motility apparatus, such as the flagella and associated axonemal proteins [86,87]. During equine semen preservation, intracellular ice crystal formation is a major cause of acrosome and organelle damage. As semen is cooled and frozen, ice crystals can develop within sperm cells, physically disrupting membranes, including the plasma membrane and the acrosome, leading to loss of acrosomal integrity and reduced fertilizing potential [88,89,90]. Cryopreservation collectively subjects equine spermatozoa to a cascade of structural insults that cumulatively compromise motility and fertilizing capacity. These detrimental effects include lipid peroxidation of cellular membranes, collapse of mitochondrial membrane potential, caspase-mediated apoptotic pathways, flagellar structural disruption, and ice crystal-induced acrosomal damage (Figure 7).

## 5. Strategies for Enhancing Equine Semen Quality

Two fundamental approaches have been extensively investigated for improving equine semen preservation quality: nutritional supplementation and the identification of potential genes that hold promise in overcoming the current barriers of equine semen cryopreservation [11,91].

Nutraceutical supplementation aims to provide essential nutrients that optimize semen parameters, including sperm concentration, motility, morphology, velocity characteristics, and overall fertility potential [92,93]. Concurrently, elucidating genetic factors in semen quality facilitates the development of targeted breeding programs and genetic selection strategies. Potential genes, such as *NME8* and *OR2AP1*, have been associated with enhanced semen preservation quality, particularly sperm motility and cryotolerance [94]. Additional genetic factors, including *CRISP3*, *SPATA1*, *INHBA*, and *ACE*, have demonstrated roles in improving sperm motility, fertilization capacity, sperm count, and structural integrity, while *PRLR* has been implicated in enhanced sperm survival [95]. The integration of these complementary strategies could significantly advance equine semen preservation techniques, resulting in improved success rates for artificial insemination and breeding programs.

### 5.1. Nutritional Supplements for Enhancing Semen Quality

Equine semen exhibits particular vulnerability to oxidative damage due to its high mitochondrial activity. Consequently, nutritional supplementation, such as vitamin E, selenium, omega-3 PUFAs, L-carnitine, and botanicals like Maca, plays a crucial role in the equine industry for improving reproductive outcomes by mitigating oxidative stress, preserving sperm membrane integrity, and enhancing motility parameters. Research efforts have focused on evaluating the effects of various dietary compounds on stallion semen quality and preservation efficacy [96]. Omega-3 polyunsaturated fatty acids (PUFAs) support membrane integrity, while L-carnitine boosts mitochondrial energy, both contributing to improved semen quality [97,98]. Botanical extracts such as Lepidium meyenii (Maca) exert positive effects on the reproductive tract and improve semen quantity and quality in mammals, as extensively reviewed by [99,100], with its beneficial actions potentially mediated through the modulation of endocrine function [101,102]. However, the effectiveness of these supplements can vary by individual, compound, and dosage, emphasizing the need for tailored supplementation programs and regular monitoring through semen analysis [96]. Several studies show the positive effects of these supplements, but variations in individual response, breed, fertility status, and study design limit comparability. Larger, standardized trials are needed to confirm efficacy and optimal dosing in diverse stallion populations. Key nutritional supplements, their mechanisms of action, and reported effects on equine semen quality are summarized in Table 2.

#### 5.1.1. Antioxidants and Vitamins

Antioxidants are essential for protecting equine sperm during cryopreservation, preserving membrane integrity, and minimizing DNA damage [103,104]. Vitamin E deficiency in stallions leads to increased abnormal sperm and reduced motility, which can be corrected with supplementation [105]. Vitamin E enhances sperm motility and reduces oxidative stress by boosting catalase (CAT) and superoxide dismutase (SOD) activity [106], Vitamin E supplementation (3000 IU/day for 14 weeks) in poor-freezer stallions showed no significant improvement in total or progressive motility of raw or frozen-thawed semen [107], while vitamin C prevents lipid peroxidation, maintaining sperm membrane stability [108]. Selenium, particularly when combined with vitamin E and L-carnitine, improves sperm motility and membrane resilience [97]. In contrast, no consistent data is available on the effects of vitamin C as a unique dietary supplement on stallions’ fertility [109], and flaxseed oil supplementation has been shown to enhance motion parameters while reducing oxidative stress [106].

Coenzyme Q10 (CoQ10) supplementation significantly improves post-thaw sperm viability [110], and vitamin A supports normal spermatogenesis and sperm function [111]. Deficiencies in these antioxidants can lead to misshapen sperm, reduced motility, and impaired fertility [105,112]. Several studies have reported no beneficial effects of antioxidant supplementation on equine semen quality [113,114]. Highlighting conflicting findings, these inconsistencies underscore the need for standardized, large-scale studies to clarify the role of individual and combined antioxidant supplements in enhancing equine semen quality.

#### 5.1.2. Minerals and Trace Elements

Minerals such as zinc (Zn) and selenium (Se) play integral roles in sperm development and function. Zinc supplementation has been demonstrated to enhance sperm motility, morphology, and viability while providing antioxidant protection, reducing oxidative stress, and maintaining sperm vitality during storage [113,115,116]. Similarly, selenium is vital for spermatogenesis and male fertility due to its potent antioxidant properties, which help preserve sperm quality [117]. These minerals contribute significantly to maintaining optimal semen parameters and protecting sperm from oxidative damage.

Trace elements, including magnesium (Mg), copper (Cu), and iron (Fe), are essential for semen quality. Magnesium levels correlate with sperm concentration and calcium levels in stallion seminal plasma [118]. Copper, a key component of antioxidant enzymes like superoxide dismutase, supports sperm motility, viability, and DNA integrity post-cryopreservation [119,120]. Iron deficiency can impair enzyme activity in seminal plasma, negatively affecting semen quality [121]. These elements collectively ensure proper sperm function and protect against oxidative damage, highlighting their importance in male reproductive health.

#### 5.1.3. Polyunsaturated Fatty Acids (PUFAs)

Polyunsaturated fatty acids (PUFAs), particularly docosahexaenoic acid (DHA) and docosapentaenoic acid (DPA), are crucial for maintaining the structural integrity, functionality, and stability of equine spermatozoa plasma membranes. Since horses, like all mammals, cannot synthesize PUFAs endogenously from saturated or monounsaturated fatty acids, these essential lipids must be supplied through dietary supplementation [122,123]. Their incorporation into sperm membranes is vital for supporting motility, membrane flexibility, and resistance to osmotic stress, especially during cooling and cryopreservation [91,124].

Studies demonstrate that DHA-enriched nutraceuticals, such as fish oil (250 g/day for 90 days) and linseed oil (150 mL/day for 60 days), significantly improve sperm motility, viability, and acrosome integrity in both fresh and frozen-thawed semen, though they may concurrently increase susceptibility to lipid peroxidation [125,126]. The lipid-modifying effects of PUFA supplementation are further evidenced by improvements in semen quality post-cooling and post-thawing, as observed with pomegranate seed oil (200 mL/day for 90 days) in Arabian stallions and flaxseed–thyme combinations (2.5% and 0.02% DMI, respectively, for 90 days) in Caspian stallions [127,128]. These enhancements are attributed to DHA’s integration into the sperm plasma membrane, which enhances progressive motility, velocity, and membrane deformability [129,130]. The effects of PUFA supplementation on semen quality remain inconsistent across studies, with some reporting marginal benefits, while others show no improvement [81]. Despite promising outcomes, the effects of PUFA supplementation remain inconsistent across studies, highlighting the need for further well-controlled trials to determine optimal dosing, duration, and effective combinations with antioxidants in diverse stallion populations.

Omega-3 fatty acids, a subclass of PUFAs, play a crucial role in enhancing stallion reproductive performance by supporting sperm membrane flexibility, elasticity, total antioxidant capacity, DNA integrity, and stability during cooling and freezing [131,132,133]. Importantly, the combined supplementation of omega-3 fatty acids with antioxidants such as L-carnitine, selenium, and vitamin E has demonstrated synergistic effects, enhancing sperm motility, lifespan, and acrosomal integrity while counteracting oxidative damage [97,134]. The effects of PUFA supplementation on semen quality remain inconsistent across studies, with some reporting marginal benefits, while others show no improvement [81]. Despite promising outcomes, the effects of PUFA supplementation remain inconsistent across studies, highlighting the need for further well-controlled trials to determine optimal dosing, duration, and effective combinations with antioxidants in diverse stallion populations.

Nutraceutical blends incorporating multiple bioactive compounds have shown broad improvements in equine semen quality across breeds. A combination of L-carnitine, selenium, vitamin E, omega-3, and omega-6 fatty acids administered over 30 weeks enhanced sperm motility, velocity, and membrane integrity [97]. Similarly, a multi-component blend containing β-carotene, lutein, lycopene, casein, selenium, DHA, and vitamins A, C, and E improved semen parameters in Colombian, Quarter Horse, Dare-Shuri, and Arabian stallions [92,135,136,137]. The benefits of these formulations are linked to their antioxidant synergy, particularly L-carnitine’s role in mitochondrial energy metabolism. Vitamin E, selenium, and zinc further bolster antioxidant defenses, preventing lipid peroxidation and preserving sperm function [138,139].

Botanical extracts, such as Lepidium meyenii (Maca), have demonstrated promising effects on stallion fertility. Studies indicate that Maca supplementation (20 g/day for 60 days) increases sperm concentration, motility, and acrosome integrity [101,140]. Additionally, Maca reduces sperm DNA fragmentation and induces favorable morphological changes, such as elongated sperm heads, which are associated with improved fertility [141]. Emerging research suggests that combining Maca with other botanicals, like Laminaria and Equisetum, may enhance post-thaw semen quality, improving total and progressive motility in cryopreserved samples [142].

Other botanical extracts, such as hemp, flaxseed, and sunflower oils, used as dietary supplements, along with microalgae, have demonstrated significant potential in enhancing semen quality and preservation across livestock species. In equines, hemp oil (rich in PUFAs) supplementation in equines (Martina Franca jacks) enhances sperm motility and morphology while improving membrane lipid stability, suggesting potential benefits for semen quality and preservation [143]. Vitamin E- and selenium-enriched microalgae enhanced sperm motility in fresh and cooled semen [144]. Similarly, flaxseed oil, rich in omega-3 alpha-linolenic acid (ALA), increased sperm motility, membrane integrity, and cryopreservation success in rams and bulls, particularly when combined with antioxidants [106,145]. Omega-6-rich sunflower oil further improved sperm fertility [146]. Stallions supplemented with a yeast-based herbal product showed no lasting improvement in semen quality or antioxidant status, with only transient benefits observed [140]. Despite promising results, evidence on botanical supplementation in stallions remains limited and inconsistent, with most data derived from non-equine species. Standardized dosing, long-term trials, and species-specific studies are needed to confirm efficacy and safety, especially under cryopreservation conditions.

Furthermore, dietary supplementation with 6% soybean lecithin improved fertility and hatchability in poultry [147], while 0.5–1.5% enhanced sperm quality in rabbits [148]. In roosters, 1% soybean lecithin combined with vitamin E improved sperm viability and membrane integrity [149]. Additionally, canola, soybean, and sunflower oils, which contain vitamin E, have been shown to positively influence fertility and semen parameters in roosters, and canola oil has been found to increase sperm count and motility in rabbits [150,151]. However, such lipid-based dietary strategies remain largely unexplored in equines, highlighting a significant research gap.

**Table 2:** Nutritional supplements enhance semen quality.

Supplement	Route of Administration/Amount/Duration	Effect on Semen Quality	References
Coenzyme Q10	Oral supplementation: 1 g/day for 4 weeks	Sperm metabolism guards against oxidative stress	[110]
Zinc	Oral supplementation: 360 1 g/day for two months	Increases average path velocity. Supports sperm morphology and motility	[113]
Zinc	Oral supplementation: 1 g/day for three months	Improved motility, viability, and membrane integrity	[115]
DHA-enriched nutraceutical	Oral supplementation: 1 g/day for 14 weeks	Increased semen DHA levels and DHA-DPA ratio	[127]
Pomegranate seed oil	Oral feed: 200 mL/day for 90 days	Improved membrane integrity and viability in cooled semen	[127]
Linseed oil	Oral feed: 150 mL/day for 60 days	Improved motility, vigor, viability, acrosome integrity, and osmotic tolerance	[125]
Fish oil + thyme	Oral supplementation: (2.5% DMI fish oil, 0.02% DMI thyme for 90 days	Enhanced total and progressive motility, sperm concentration, and membrane integrity	[128]
Vitamin E, selenium, L-carnitine, and fatty acids	Oral supplementation: 50 mL/day for two months	Higher progressive motility, membrane and acrosomal integrity	[97]
Plasmolyzed herbal yeast	Oral dose: 0.06 mL/kg body weight/day/70 days	Antioxidant status increased	[140]
Lepidium meyenii (Maca) powder	Oral dose: 20 g/day/60 days	Improved total and progressive motility, as well as acrosome integrity	[110]
Encapsulated TE (ETE)	Oral/1.5–3 g/16 weeks	Improved testicular length, sperm concentration, and reduced non-progressive spermatozoa	[152]
Hemp oil (PUFAs)	Oral supplementation: 20 mL/day/90 days	Increased sperm motility and normal morphology, reduced gel-free semen volume, and lower peroxidation	[143]
Flaxseed oil	Oral supplementation: 24 g/kg diet/60 days	Improved sperm motility, membrane integrity, and fertilization capacity. Reduced oxidative stress	[106,145]
Sunflower oil (SFO)	Oral supplementation: 35 g/day/for 15 weeks	Enhanced sperm motility and progressive motility Improved sperm fertility	[146]

### 5.2. Screening of Potential Candidate Genes Associated with Semen Quality in Equines

The identification of genetic determinants influencing sperm quality and cryotolerance represents a critical area of investigation in equine reproductive biology. Elucidating the genetic architecture underlying these traits holds significant promise for enhancing breeding efficiency, optimizing preservation protocols, and addressing persistent challenges in equine reproductive management [95]. Recent advances in genomic methodologies, particularly genome-wide association studies (GWASs), have facilitated the identification of several candidate genes implicated in various aspects of semen quality [94,153,154]. Consistently, potential genes have been widely documented for their association with semen-quality traits [155].

Genomic investigations have revealed specific loci associated with post-thaw sperm characteristics, including the olfactory receptor genes *OR2AP1* and *OR6C4*, as well as the *NME8* gene, which demonstrates significant associations with the maintenance of sperm motility following cryopreservation in stallions [94]. These findings suggest the potential involvement of olfactory receptor-mediated signaling pathways in sperm function and cryotolerance, offering novel insights into the molecular mechanisms governing post-thaw semen quality.

A comprehensive GWAS analysis conducted on German warmblood stallions has identified 29 single-nucleotide polymorphisms (SNPs) distributed across 12 chromosomes that exhibit statistically significant associations with diverse semen quality parameters. Genes such as *HERC4*, *MIER1*, *NEGR1*, *CTNNA3*, *C1QTNF7*, and *CRISP1* have been implicated in modulating critical attributes, including sperm concentration, semen coagulation characteristics, progressive motility, zona pellucida binding capacity, and gel-free semen volume [156]. Additionally, genes encoding antioxidant enzymes and molecular chaperones, such as superoxide dismutase 1 (*SOD1*), chaperonin containing TCP1 subunit 8 (*CCT8*), and sirtuin 1 (*SIRT1*), have demonstrated significant associations with various semen-quality traits [156,157,158]. These highlight the importance of oxidative stress management and protein folding in maintaining sperm integrity.

The cysteine-rich secretory protein 3 (*CRISP-3*) gene has emerged as a particularly significant modulator of stallion semen quality, exerting positive effects on sperm morphology, motility parameters, cellular viability, and membrane structural integrity [159]. In contrast, the FK506 binding protein 6 (*FKBP6*) gene has been associated with impaired acrosomal reaction kinetics and reduced conception rates in Hanoverian stallions, illustrating the potentially deleterious effects of specific genetic variants on fertility outcomes [160,161]. Other critical genes, including heat shock protein 1 (*HSP1*), *CRISP3*, and kallikrein-related peptidase 1E2 (*KLK1E2*), have been implicated in fundamental reproductive processes such as sperm motility regulation, DNA structural stabilization, capacitation, and gamete fusion [162].

The microtubule-associated protein 1 light chain 3 (*LC3*) gene, an integral component of the autophagy pathway, plays a crucial role in maintaining sperm viability during preservation. Cooling and cryopreservation procedures trigger autophagy-related processes, resulting in the conversion of LC3-I to LC3-II, which serves as a molecular indicator of autophagy activation [163]. Similarly, the A-kinase anchoring protein 4 (*AKAP4*) gene, particularly its precursor pro*AKAP4*, has been established as a critical determinant of equine semen quality through its significant influence on both total and progressive sperm motility parameters [164]. The insulin-like growth factor 1 (*IGF1*) gene has also demonstrated associations with ejaculate volume, sperm concentration, and motility characteristics in stallions [165]. However, the precise mechanisms and interactive roles of these genes in equine semen preservation remain unclear, requiring comprehensive functional studies.

Additional genetic factors, including phospholipase C zeta (*PLCζ*), angiotensin-converting enzyme (*ACE*), sperm protein 17 (*SP17*), and follicle-stimulating hormone beta subunit (*FSHB*), contribute to equine semen quality and fertility by modulating key reproductive parameters such as pregnancy rates and breeding values [166,167]. The spermatogenesis-associated protein 1 (*SPATA1*) gene has been identified as a significant contributor to sperm head morphology, enhancing stallion fertility through improved pregnancy rates and breeding values [168]. Finally, the sodium voltage-gated channel alpha subunit 8 (*SCN8A*) gene, localized to the flagellum and pericentriolar region of spermatozoa, regulates sperm motility through ion channel-mediated mechanisms [169]. Therefore, to fully realize the potential of genomic selection and biomarker development in equine reproduction, large-scale, multi-breed studies with standardized semen quality assessments and functional gene validation are essential. A summary of potential genes associated with semen-quality traits in equines is provided in Table 3.

**Table 3:** Potential candidate genes related to sperm-quality traits in equines.

Gene Symbol	Breed	Functional Role	Effect of Semen-Quality Trait	References
*NME8*	Stallion	Sperm structure and motility	Essential for sperm tail formation and motility	[94,160,161]
*OR2AP1*	Stallion	GPCR signaling, sperm motility regulation, cryotolerance	Improved post-thaw sperm motility	[94]
*OR6C4*	Stallion	GPCR-mediated sperm motility signaling	Associated with sperm motility	[94]
*HERC4*	German Warmblood stallions	Protein degradation, spermatogenesis regulation	Potential impact on sperm motility and fertility	[156]
*MIER1*	German Warmblood stallions	Potential role in spermatogenesis	Associated with gel-free semen volume	[156]
*NEGR1*	German Warmblood stallions	Sperm concentration regulation	linked to sperm concentration	[156]
*CTNNA3*	German Warmblood stallions	Spermatogenesis and sperm motility	Associated with progressive sperm motility	[156]
*C1QTNF7*	German Warmblood stallions	Sperm motility	Linked to progressive sperm motility	[156]
*CRISP1*	Stallions	Protects live sperm from PMN binding and phagocytosis during breeding-induced endometritis	Positive correlation with fertility (higher CRISP3 levels linked to improved pregnancy rates)	[170,171,172,173]
*SOD1*	Stallion	Primary antioxidant defense in spermatozoa protects against oxidative stress during cryopreservation	Reduction after cryopreservation correlates with increased oxidative damage and lipid peroxidation (4-HNE)	[157]
*SIRT1*	German Warmblood	Maintains mitochondrial function	Influence sperm production and quality	[156,174,175]
*FKBP6*	Hanoverian	Conception rates Meiosis (chromosome pairing)—Acrosome reaction (AR)	Impaired acrosome reaction, conception rate	[160,161]
*CRISP3*	Stallions	Morphology	Improving motility, vitality, morphology, and membrane integrity	[161,176]
*HSP1*	Horse	Acts as a molecular chaperone (protects against oxidative stress)	Role in sperm motility, viability, and oviductal sperm reservoir formation	[162]
*KLK1E2*	Horse	Seminal plasma protein, androgen-regulated	Involved in sperm membrane integrity and function, linked to capacitation	[162]
*IZUMO4*	Horse	Gamete recognition, acrosomal protein	Potential role in fertilization. Involved in pre-fertilization stages (e.g., acrosome reaction)	[162]
*LC3* *MAP1LC3*	Stallion (Andalusian horses)	Autophagy marker, acrosome reaction regulation	Enhances sperm capacitation and acrosome reaction, critical for membrane remodeling during fertilization	[132,163]
*AKAP4* *proAKAP4*	Stallions	Fibrous sheath assembly, sperm flagellar structure, motility regulation, and capacitation	Total sperm motility Progressive motility Sperm viability	[164]
*IGF1*	Horse	Sperm production and metabolic activity	Relationship between IGF1 concentrations and sperm motility	[165]
*PLCζ*	Criollo stallions	Signaling (calcium release)	Egg activation, calcium signaling, embryonic development, and expression correlate with sperm motility	[167,177]
*ACE*	Hanoverian stallions	Affects sperm migration, zona pellucida binding	Associated with an embryonic component of breeding values (BVs) and pregnancy rate per oestrus (PRO)	[166]
*SP17*	Hanoverian stallions	Cell adhesion and fertilization	Association with embryonic and paternal components of BVs	[166]

Note: *NME8* (non-metastatic melanoma protein 8), *OR2AP1* (olfactory receptor family 2 subfamily AP member 1), *OR6C4* (olfactory receptor family 6 subfamily C member 4), *HERC4* (*HECT* and *RLD* domain-containing E3 ubiquitin protein ligase 4), *MIER1* (mesoderm induction early response protein 1), *NEGR1* (neuronal growth regulator 1), *CTNNA3* (catenin alpha 3), *C1QTNF7* (C1q and tumor necrosis factor-related protein 7), *CRISP1* (cysteine-rich secretory protein 1), *SOD1* (superoxide dismutase 1), *SIRT1* (sirtuin 1), *FKBP6* (FKBP prolyl isomerase 6), *CRISP3* (cysteine-rich secretory protein 3), *HSP1*, *KLK1E2* (kallikrein-1-related peptidase E2 pseudogene), *IZUMO4* (IZUMO family member 4), *LC3* (*MAP1LC3B*) (microtubule-associated protein 1 light chain 3 beta), *AKAP4* (pro*AKAP4*) (A-kinase anchoring protein 4), *IGF1* (insulin-like growth factor 1), *PLCζ* (phospholipase C zeta), *ACE* (angiotensin-converting enzyme), and *SP17* (sperm auto antigenic protein 17).

### 5.3. Advances in Semen Extender Formulations and Nutraceutical Integration

Recent advancements in semen extender formulations have significantly improved equine semen preservation outcomes. Modified INRA-82 (m-INRA) has demonstrated superior performance over traditional extenders like modified Kenney’s (m-Kenney) and Equi-Pro^®^ (Inertia Technology, Enschede, The Netherlands), yielding higher post-thaw motility (52.5%) and better preservation of membrane, acrosomal, and mitochondrial function [178,179,180]. Defined milk protein-based extenders (e.g., INRA96 and EquiPro) outperform skim milk-based options for liquid storage, while novel formulations like HF-20 (containing raffinose and glucose) and cross-species adaptations such as Steridyl^®^ (Minitube, Tiefenbach, Germany) (originally for ruminants) show promising cryoprotective effects [181,182,183]. Table 4 compares these extenders, highlighting their varying efficacy in enhancing semen quality metrics, including motility, membrane integrity, and oxidative stress resistance.

The incorporation of nutraceuticals into extenders has further optimized semen preservation. Natural antioxidants, like gallic acid (50 µg/mL), mitigate oxidative stress, while L-carnitine (1–2 mM) preserves motility and membrane integrity during chilled storage [136,184,185,186,187]. Coenzyme Q10 (1 µM) combined with α-tocopherol (5 mM) reduces lipid peroxidation and enhances sperm viability, whereas nicotinic acid (20 mM) improves cooled semen quality by reducing DNA fragmentation; however, higher doses (40 mM) impair motility [188,189]. Soybean lecithin-based extenders also emerge as effective alternatives to egg yolk, maintaining post-thaw viability, motility, and fertilizing potential [190,191]. Additional additives like caffeine (2–4 mM) and taurine (25 mM) synergistically sustain progressive motility for up to 96 h [192].

Optimized cooling protocols, such as storage at 5 °C, extend semen viability to 40 h while maintaining motility and DNA integrity [193]. Low-dose ozone (2–15 µg/mL) enhances chilled sperm function, though higher concentrations (30–60 µg/mL) are detrimental [194]. Sucrose (50–100 mM) also serves as a viable cryoprotectant, improving post-thaw motility and mitochondrial function [195]. These innovations, combined with advanced extender formulations and nutraceuticals, represent a holistic approach to equine semen preservation, bridging genetic, nutritional, and biotechnological strategies [196]. Collectively, advancements in extender formulations and nutraceutical integration demonstrate notable improvements in equine semen preservation; however, optimizing synergistic combinations and validating long-term fertility benefits remain critical research priorities.

**Table 4:** Advances in semen extender formulations and nutraceutical integration enhance semen quality preservation.

Supplement	Supplementation Method and Concentration	Effect on Semen Quality	References
Gallic Acid (GA)	50–100 µg/mL in Tris-based extender; frozen at −196 °C	Improved progressive motility and DNA integrity; increased post-thaw total motility and semen concentration, membrane integrity, testosterone, motility, and fertility	[184,185,186,187]
L-Carnitine (LC)	1–2 mM in skimmed milk extender; 48 h at 5 °C	Preserves motility and plasma/acrosomal membrane integrity; reduces oxidative stress.	[136]
Sucrose	50–100 mM in skimmed milk–egg yolk extender	Highest plasma membrane integrity, best acrosomal preservation, superior mitochondrial potential, improved wobble (WOB)	[195]
CoQ10 (C1)	Added to semen extender (175–700 µM)/Frozen storage	Improved membrane integrity, stability, and pregnancy	[188]
Soybean Lecithin	Tris-based extender with 4% glycerol	Better protection against membrane damage and lipid peroxidation Preserved DNA and acrosome integrity	[190,191]
Nicotinic Acid (NA)	Added to semen extender at 10, 20, and 40 mM	Improved viability and membrane and acrosome integrity, reduced DNA fragmentation, decreased lipid peroxidation and ROS/RNS	[189]

## 6. Conclusions

Improving equine semen quality is essential for maximizing reproductive efficiency and ensuring the sustainable management of equine populations. This review highlights the multifaceted challenges associated with semen preservation, including individual variability, biological contamination, sensitivity to cryopreservation, oxidative stress, cold shock, epigenetic alterations, and limited understanding of molecular mechanisms. This review highlights the multifaceted nature of these challenges and the need for comprehensive solutions.

Despite these challenges, significant progress has been made using nutritional supplements. Antioxidant supplementation with vitamin E, selenium, L-carnitine, and polyunsaturated fatty acids has shown promise in mitigating oxidative stress and enhancing sperm parameters. Additionally, advanced extender formulations and optimized cooling protocols can significantly enhance post-thaw sperm viability, motility, and DNA integrity. Moreover, genomic insights into candidate genes like NME8, CRISP3, OR2AP1, and SPATA1 are associated with improved semen quality and cryotolerance.

Despite recent advances, the field still faces considerable limitations. Current extenders, while improved, are not universally effective across stallions due to high individual variability. Similarly, many nutraceutical interventions show inconsistent results, underscoring the need for individualized supplementation protocols. The role of epigenetic changes and RNA integrity in sperm functionality post-thaw remains underexplored. Moreover, the integration of semen-quality biomarkers into routine cryopreservation assessments is limited.

Future research should prioritize stallion-specific cryopreservation protocols using genomic and proteomic profiling to address individual variability in semen quality. Functional studies on candidate genes like NME8 and CRISP3, along with multi-omics approaches, can help identify reliable biomarkers for sperm cryotolerance. Innovations in antioxidant delivery and standardized trials on nutritional supplements are needed to improve and validate their effectiveness. Additionally, studies on epigenetic changes, offspring fertility outcomes, cost-effective extenders, and non-antibiotic antimicrobial strategies will support more efficient and sustainable equine semen preservation.

## Figures and Tables

**Table 1:** Factors affecting equine semen preservation quality.

Factors	Affecting Semen-Quality Traits	References
Environmental Conditions	Different environmental conditions, such as humidity, temperature, and seasonal changes, cause stress to cells, which, in turn, decreases sperm motility and viability.	[69]
Collection Techniques	Techniques such as the use of artificial vaginas can influence semen volume, concentration, and the composition of seminal plasma.	[70]
Bacteria	Bacterial contamination reduces sperm motility and semen shelf life.	[71]
Cryopreservation	The formation of ice crystals during freezing can harm sperm cells, affecting their head shape and size, and disrupt their movement.	[72,73]
Oxidative Stress	Oxidative stress leads to membrane damage, DNA fragmentation, and protein degradation in stored semen.	[18]
Osmotic Stress	Hypertonic media change sperm metabolism, leading to reduced motility and viability.	[74]
Apoptosis	It causes damage to membranes, leads to DNA fragmentation, and results in the formation of apoptotic bodies, ultimately decreasing sperm fertility.	[20]
Stallion Age	Semen quality declines in very young (<3 years) and older stallions (>11 years).	[75]
Centrifugation Parameters	High force and prolonged duration can result in sperm loss when removing the supernatant.	[76]
Lack of Standardization	Inconsistent laboratory techniques lead to varying assessments of semen quality.	[70]
Seasonal Effects	Affect motility, membrane integrity, and sperm DNA fragmentation	[67]

## Data Availability

No new data created.
